# Behavioral intention to use electronic cigarettes in the Philippines: The role of social influence, knowledge, price and health impact

**DOI:** 10.1371/journal.pone.0318630

**Published:** 2025-02-06

**Authors:** Zachariah John A. Belmonte, Yogi Tri Prasetyo, Pamela Eyre R. Victoria, Maela Madel L. Cahigas, Reny Nadlifatin, Ma. Janice J. Gumasing

**Affiliations:** 1 School of Industrial Engineering and Engineering Management, Mapúa University, Manila, Philippines; 2 School of Graduate Studies, Mapúa University, Manila, Philippines; 3 Mechanical Engineering & Allied Department, Technological University of the Philippines, Manila, Philippines; 4 International Bachelor Program in Engineering, Yuan Ze University, Chung-Li, Taiwan; 5 Department of Industrial Engineering and Management, Yuan Ze University, Chung-Li, Taiwan; 6 National Institute of Geological Sciences, University of the Philippines, Quezon City, Diliman, Philippines; 7 Department of Information Systems, Institut Teknologi Sepuluh Nopember, Kampus ITS Sukolilo, Surabaya, Indonesia; 8 Department of Industrial and Systems Engineering, Gokongwei College of Engineering, De La Salle University, Manila, Philippines; University of Saskatchewan, CANADA

## Abstract

Electronic cigarettes or e-cigarettes have gained significant popularity as an alternative to traditional cigarettes, yet limited research has examined the factors influencing their adoption, particularly in developing nations like the Philippines, where usage is rising. This study investigates the behavioral drivers of e-cigarette use, with a particular focus on the role of knowledge, alongside social influence, perceived price impact, perceived health impact, and perceived usefulness. Using purposive sampling, 310 valid responses were collected from current e-cigarette users, traditional cigarette users, or individuals with prior experience with these products. A structured questionnaire with 21 indicators was administered, and data were analyzed using Partial Least Square-Structural Equation Modeling (PLS-SEM). The findings reveal that knowledge is the strongest predictor of behavioral intention, highlighting the critical role of informed awareness about the risks and impacts of e-cigarettes in shaping user decisions. Social influence, perceived price impact, and perceived health impact also significantly influence behavioral intention, demonstrating the interconnectedness of cognitive, social, and economic factors. Interestingly, perceived usefulness did not have a significant effect, challenging assumptions about the importance of functional benefits in driving e-cigarette adoption. These results underscore the importance of education and awareness campaigns in addressing misconceptions about e-cigarettes. Policymakers, regulators, and health professionals should prioritize knowledge-driven interventions to empower individuals to make informed decisions and mitigate e-cigarette use, particularly among younger and economically vulnerable populations.

## 1. Introduction

Electronic cigarettes (e-cigarettes) have gained popularity as an alternative to traditional cigarettes, driven by their perceived health benefits and reduced social stigma compared to conventional smoking [1]. Developed in 2003 by Chinese pharmacist Hon Lik as a smoking cessation tool [2], e-cigarettes vaporize a liquid composed of propylene glycol, glycerol, flavorings, and often nicotine. This vaporization process mimics the sensory experience of smoking without combustion, a feature marketed as a safer and socially acceptable alternative [3]. However, growing evidence highlights significant health risks associated with e-cigarette use, including severe lung injuries, nicotine addiction, and chronic respiratory diseases [4,5].

Globally, e-cigarette usage has increased substantially since its introduction in 2006. In the United States, 15.9% of adults have reported using e-cigarettes, with adoption rates highest among individuals aged 18–44 years [6]. Similarly, 21.9% of conventional cigarette users in the United Kingdom have transitioned to e-cigarettes [7]. In Southeast Asia, adolescent e-cigarette usage ranges from 3.3% to 11.8% [8]. As of 2021, an estimated 82 million people worldwide use vapes, marking a 17% increase from the previous year [9]. These global trends highlight the need for localized studies to understand behavioral drivers and public health implications.

In the Philippines, e-cigarettes were introduced in 2010 and have since become a pressing public health concern. The country accounts for approximately 2.7 million vape users, representing 3% of the global total [9]. Among Filipino youth aged 13–15, e-cigarette use is particularly concerning, with the 2019 Global Youth Tobacco Survey (GYTS) reporting that 14.1% are current e-cigarette users, exceeding the 12.5% prevalence of other tobacco products. Additionally, 24.5% of students in this age group reported having tried e-cigarettes, and 37% of youth who smoke indicated easy access to tobacco products despite age restrictions [10].

Economic projections further emphasize the significance of e-cigarettes in the Philippines. The e-cigarette market is projected to generate US$252.4 million in revenue by 2024 [11]. However, public awareness campaigns and regulatory frameworks remain underdeveloped, underscoring the urgent need for research into consumer behavior and health perceptions to inform effective interventions.

This study addresses this critical gap by investigating the behavioral drivers of e-cigarette use in the Philippines. Specifically, it examines the roles of social influence, price, perceived health impact, usefulness, and knowledge in shaping behavioral intentions. Employing Structural Equation Modeling (SEM), the research provides a comprehensive framework for understanding e-cigarette adoption. The findings aim to support evidence-based policymaking, public health initiatives, and regulatory strategies tailored to the Philippine context.

## 2. Review of related literature

### 2.1 The role of social influence

Social influence plays a critical role in shaping individuals’ decisions to adopt e-cigarettes, particularly among younger demographics. Peer behavior and societal norms often normalize e-cigarette use, making it more appealing to those seeking acceptance or conformity [3]. Social media further amplifies this effect by portraying e-cigarettes as trendy and socially acceptable, particularly through influencers and targeted advertising campaigns [12]. Research indicates that these platforms contribute to the glamorization of vaping, creating environments where e-cigarette use is perceived as modern and socially desirable [8]. This normalization is especially concerning in the Philippine context, where youth accessibility and peer dynamics significantly drive adoption rates [10].

### 2.2 The role of perceived price impact

Perceived price impact is a significant factor influencing e-cigarette adoption, particularly in low- and middle-income countries such as the Philippines. Consumers often perceive e-cigarettes as more affordable than traditional tobacco products, a belief reinforced by frequent promotional discounts and price incentives [9]. This affordability lowers financial barriers to entry, especially among younger users and those with limited purchasing power. In the Philippines, the e-cigarette market’s projected revenue of US$252.4 million by 2024 [11] reflects the increasing accessibility and appeal of these products. However, inconsistencies in the enforcement of price regulations and tax measures allow e-cigarettes to remain relatively affordable for vulnerable groups, including youth and low-income populations [10]. This highlights the need to address perceived price-related drivers to mitigate the growing prevalence of e-cigarette use in the country.

### 2.3 The role of perceived health impact

The perceived health benefits of e-cigarettes are a significant motivator for their adoption. E-cigarettes are often marketed as less harmful alternatives to traditional smoking due to their lack of combustion and lower levels of harmful chemicals like tar [3]. This perception has led to widespread beliefs that e-cigarettes pose fewer health risks, making them an attractive option for those seeking harm reduction or smoking cessation [2]. However, emerging evidence contradicts these claims, highlighting the presence of harmful substances in e-cigarette aerosols that contribute to respiratory illnesses and long-term health complications [4]. In the Philippines, where public awareness campaigns are limited, misconceptions about the health impacts of e-cigarettes persist, further complicating efforts to regulate their use and educate the public effectively [10].

### 2.4 Perceived usefulness of e-cigarettes

The perceived usefulness of e-cigarettes plays a significant role in their adoption, particularly as they are often marketed as a harm-reduction tool. This perception stems from the belief that e-cigarettes are less harmful than traditional cigarettes because they do not involve combustion, a process that produces tar and other harmful byproducts [3]. Studies indicate that users often consider e-cigarettes an effective smoking cessation aid, with some individuals perceiving them as a stepping stone toward quitting traditional cigarettes altogether [2].

## 3. Conceptual framework and hypothesis development

**Fig 1:**
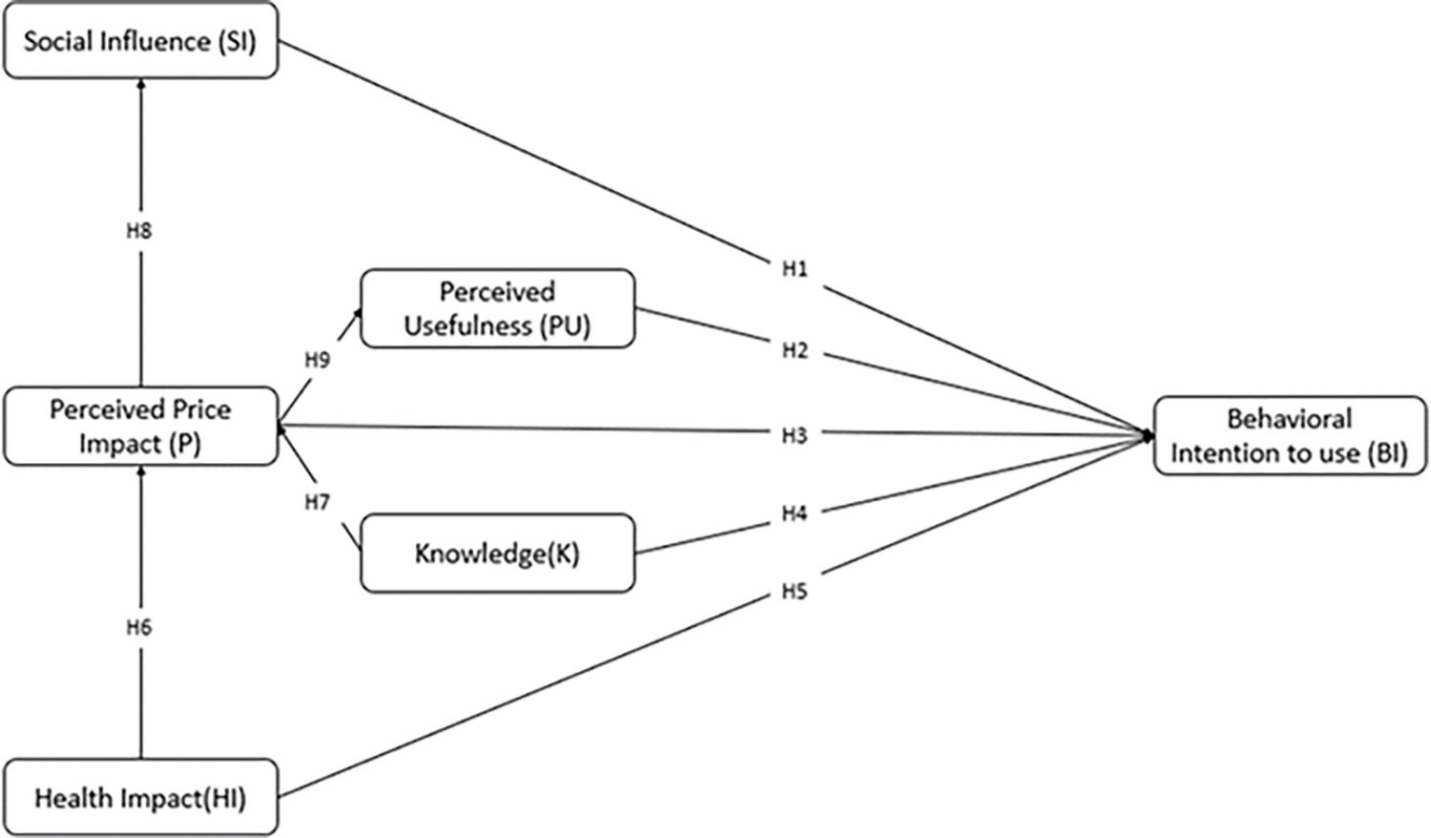
Conceptual research framework.

Fig 1 represents the conceptual framework of this study. There are one exogenous variable (Health Impact) and five endogenous (Perceived Price Impact, Social Influence, Health Impact, Perceived Usefulness, Knowledge, and Behavioral Intention to use) variables proposed in this study.

Social influence plays a critical role in shaping behavioral intentions toward e-cigarette use. It refers to the impact of individuals’ social circles, such as friends, family, and peers, on their attitudes and decision-making processes. Studies have shown that individuals who perceive positive attitudes toward e-cigarette use from their close social networks are more likely to develop favorable intentions to use e-cigarettes [13]. Conversely, those who believe their friends or family hold negative views about e-cigarette use exhibit reduced behavioral intentions and lower self-efficacy in adopting or continuing the behavior [14]. Social norms and expectations within social groups significantly drive e-cigarette adoption, particularly in environments where vaping is seen as acceptable or even desirable. These influences are especially pronounced among young adults and adolescents, who may be more susceptible to peer pressure and social conformity.

***H1*. *Social Influence [SI] had a significant relationship with Behavioral Intention to use [BI]*.**

The research examined in this article underscores electronic cigarettes as a notably safer alternative to traditional tobacco cigarettes. Designed to mitigate tobacco-related health risks, electronic cigarettes aim to reduce cigarette consumption and smoking rates. In comparison to placebo electronic cigarettes and nicotine patches, electronic cigarettes appear to offer effective assistance to smokers who struggle to quit completely, aiding in the reduction of cigarette consumption. However, it’s important to acknowledge that certain limitations affect the certainty of this finding. Nevertheless, electronic cigarettes are likely to surpass traditional pharmacotherapy methods for smoking cessation [15]. It’s worth noting that this research is further limited by the absence of biochemical evaluations to measure the actual reduction in cigarette intake. Currently, there is no conclusive evidence suggesting that short-term electronic cigarette use poses significant health risks [16]. Thus, the researchers hypothesized as follows:

***H2*. *The perceived usefulness of e-cigarettes as an alternative to traditional cigarettes [PU] had a significant relationship with Behavioral Intention to use [BI]*.**

Studies suggest that electronic cigarettes (e-cigarettes) have the potential to serve as substitutes for traditional cigarettes and may contribute to a reduction in tobacco consumption. However, it’s important to note that the availability and relative cost of e-cigarettes could influence smokers’ decisions to quit completely. Therefore, policymakers should carefully consider maintaining a consistent price differential between e-cigarettes and traditional cigarettes [17]. Even with a uniform national cigarette tax, cigarette prices vary significantly across the country, ranging from 14 to 40 pesos per pack based on 2009 data. This wide price disparity persisted even after the implementation of the sin tax reform, which aimed to standardize the tax structure to a unitary excise duty of 30 Philippine pesos per pack. As of 2015, average prices ranged from 29 to 63 Philippine pesos per pack [18]. Furthermore, the cost of first-generation e-cigarettes in the United States remains relatively affordable. Expenditure on e-cigarettes appears to be closely linked to consumer behavior regarding e-cigarette use. Understanding the potential relationships between spending at vape shops and consumer behavior is essential, especially in the context of proposed regulations for e-cigarette sales [19]. Thus, we hypothesized as follows:

***H3*. *Perceived Price Impact[P] had a significant relationship with Behavioral Intention to use [BI]***

Physicians engaged in discussions regarding various e-cigarette-related matters, encompassing their perceived level of harm in comparison to traditional cigarettes and nicotine replacement therapies (NRT), as well as emerging public health concerns associated with e-cigarettes. A significant portion of medical professionals held the belief that e-cigarettes presented greater risks than nicotine replacement therapies such as nicotine patches and gum but were still considered less harmful than conventional cigarettes [20]. Given these perspectives, public health authorities should prioritize the effective dissemination of information concerning the advantages and disadvantages of e-cigarettes. This communication effort should employ a multichannel approach and be supported by ongoing monitoring of the industry’s marketing strategies, particularly in anticipation of impending legislation [21]. Thus, the researchers hypothesized as follows:

***H4*. *Knowledge [K] had a significant relationship to Behavioral Intention to use [BI]*.**

The promotion and use of e-cigarettes have sparked debate and controversy. This study introduces a framework aimed at delineating and synthesizing trends in combustible cigarette use to determine the cumulative extent of harmful health consequences for users. The research commences by examining toxicity and its implications for public health [22]. While transitioning to e-cigarettes may offer relief from oral symptoms for regular smokers, findings from this review suggest a wide array of oral health complications may be associated with e-cigarette use [23]. However, it’s important to note that compared to conventional cigarette smokers, the available scientific evidence regarding the human health impacts of e-cigarettes remains limited. While e-cigarette aerosols may contain fewer toxins than tobacco smoke, studies assessing whether e-cigarettes are indeed less harmful than traditional cigarettes yield uncertain results [24]. Thus, we hypothesized as follows:

***H5*. *Health Impact [HI] significantly affected Behavioral Intention to use [BI]*.**

The actual impact of e-cigarettes on population health hinges on a complex interplay of factors, including their influence on smoking initiation and cessation, levels of dual usage (simultaneous use of traditional cigarettes and e-cigarettes), and product toxicity. The long-term health risks associated with e-cigarettes persist [25]. Currently, there is no definitive evidence on how changes in relative pricing, such as taxing e-cigarettes and raising tobacco prices, may affect e-cigarette sales in the EU. Proposed regulations aim to restrict the nicotine content in e-cigarettes and refill containers, mandate health warnings and child-proof packaging, and prohibit advertising unless companies obtain clearance and authorization to market their products as medications [26]. The Food and Drug Authority (FDA) can mitigate the adverse health effects of tobacco by prohibiting false or deceptive health claims and hazardous components. Additionally, the FDA has the authority to regulate the voltage of e-cigarette batteries to prevent them from exceeding dangerous limits. While the FDA lacks the power to levy taxes directly, it can indirectly influence prices by enforcing marketing and product requirements that drive up manufacturing costs for businesses [27]. Thus, the researchers hypothesized as follows:

***H6*. *Health Impact [HI] significantly affected Perceived Price Impact [P]*.**

Research indicates that exposure to tobacco marketing can lead to misconceptions and misinformation among the public regarding the risks associated with tobacco use. This is partly due to marketing messages that portray electronic cigarettes as more appealing and less harmful than traditional tobacco cigarettes, leading consumers to believe that they are a safer option [28]. A recent systematic review has revealed that many adolescents are unaware that e-cigarette liquid often contains nicotine, a substance also found in combustible cigarettes and other tobacco products. Furthermore, studies demonstrate that a majority of adolescents perceive e-cigarette use as significantly less harmful than smoking traditional cigarettes or using other tobacco products [29]. The long-term health effects of e-cigarettes remain largely uncertain, given their relatively recent development. Consequently, ongoing monitoring of e-cigarette use is imperative in light of the potential diverse effects associated with them. Similar to other nicotine and tobacco products, several factors can influence an individual’s inclination to use e-cigarettes. Previous research has indicated that exposure to e-cigarette advertising and a reduced perception of harm are correlated with an increased likelihood of usage [30]. Thus, the researchers hypothesized as follows:

***H7*. *Knowledge [K] had a significant effect on Perceived Price Impact [P]*.**

One fundamental principle of the theory of social comparison processes suggests that when an individual encounters multiple seemingly unrelated sources that all agree on a particular matter, and there is no apparent explanation for their consensus, it becomes reasonable to infer that these sources are correct. Consequently, individuals may be influenced by this perceived collective correctness [31]. However, this rule can sometimes be overgeneralized, leading to undue influence from others even when they are incorrect, as originally proposed by Festinger in 1954. An established empirical finding in this context is that a majority tends to exert more influence than a minority [32]. In the realm of consumer behavior, individuals who choose to repurchase the same brand or product typically do so because they have had a positive prior experience or because their expectations have been consistently met. Repeat purchases are often considered the most pivotal factor contributing to a company’s overall profitability, as highlighted by Reichheld [33]. Thus, the researchers hypothesized as follows:

***H8*. *Perceived Price Impact [P] significantly affected Social Influence [SI]*.**

Perceived usefulness is a crucial metric employed to gauge an individual’s confidence in utilizing a system to enhance their performance. The belief that information systems are valuable leads to their usage, and conversely, the system’s usage reinforces this belief. Moreover, consumer confidence is closely linked to the level of convenience and simplification provided by financial transactions in daily life [34]. Price is defined as the monetary amount charged for a product or service, representing the value consumers exchange for the benefits associated with owning or using said product or service [35]. Product quality, on the other hand, pertains to a product’s ability to deliver results or performance that align with or exceed customer expectations. Quality is a critical competitive factor for businesses aiming to satisfy their customers. While some may believe that quality comes at a higher price, companies that manage to deliver quality products at a reasonable cost can effectively meet customer demands and expectations [36]. Thus, the researchers hypothesized as follows:

***H9*. *Perceived Price impact [P] had a significant effect on Perceived Usefulness [U]*.**

## 4. Methodology

This study was approved by the Mapúa University Research Ethics Committee (FM-RC-22-03). An online consent form was signed by each participant before data collection. Due to stringent health protocols in place during the COVID-19 pandemic, the research was conducted through a survey questionnaire distributed via Google Forms, ensuring accessibility for participants. Data collection was carried out from September 1, 2022, to December 1, 2022, and the survey was disseminated through various social media platforms to target participants with prior knowledge of electronic cigarette usage. Before participating, all respondents were provided with detailed information about the study’s objectives and purpose. Explicit consent was obtained for the use of their personal information, in full compliance with the Philippine Data Privacy Act [37]. This ensured that all data was handled confidentially and responsibly throughout the research process. For this analysis, the data will be examined as a whole, without separating respondents by specific generational cohorts.

### 4.1. Questionnaire design

The research instrument was designed to assess the impact of Social Influence (SI), The perceived usefulness of e-cigarettes as an alternative to traditional cigarettes (PU), Perceived Price Impact (P), Knowledge (K), and Health Impact (HI) on the participants’ behavioral intention to use (BI) through different constructs as shown in Table 1. The Likert 5-point was used to standardize the extent of the responses with the assumed latent variables.

**Table 1:** Research instruments.

Latent Variable	Items	Construct	Reference
Social Influence (SI)	**SI1**	When compared to traditional cigarettes, e-cigarettes are more socially acceptable.	Rom et al. [3]
	**SI2**	The electronic cigarette is a new phenomenon that is gaining popularity among smokers all over the world.	Caponnetto et al. [38]
	**SI3**	I use electronic cigarettes because people around me believe it helps relieve stress.	Kong et al. [39]
Perceived usefulness (PU)	**U1**	Electronic cigarettes may be seen as more convenient than traditional cigarettes.	Jiang et al. [40]
	**U2**	E-cigarettes are a more effective alternative to smoking.	Farsalinos & Polosa [41]
	**U3**	The use of e-cigarettes means that these devices are designed to supply nicotine.	Barbeau et al. [42]; Polosa et al. [43]
Perceived Price Impact (P)	**P1**	Most people believed that vaping was less expensive than smoking.	McQueen et al. [44]
	**P2**	E-cigarettes can discourage smokers from quitting smoking altogether when cigarette prices rise.	E-Grace et al. [45]
	**P3**	The current price of e-cigarettes is generally much higher than flammable cigarettes.	Liber et al. [46]
Knowledge (K)	**K1**	People who smoke can reduce their smoking and quit using e-cigarettes.	Hartmann-Boyce et al. [47]
	**K2**	Electronic cigarettes hurt human health.	Jiang et al. [40]; Aghar et al. [48]
	**K3**	The e-cigarette aerosols that users inhale may contain harmful and potentially harmful substances.	Rosy [49]
Health Impact (HI)	**HI1**	E-cigarettes are probably less harmful than smoking cigarettes.	Etter & Bullen [50]
	**HI2**	The use of e-cigarettes is associated with the same adverse health effects as traditional cigarettes.	Hernandez et al. [51]
	**HI3**	Electronic cigarettes are more addictive than smoking cigarettes.	Jankowski et al. [52]
	**HI4**	E-cigarettes release a variety of toxic chemicals to the lungs.	Gotts et al. [53]
	**HI5**	E-cigarettes can damage other systems such as the liver, kidneys, and nervous system.	Cao et al. [54]
	**HI6**	Acute pulmonary injury is most likely due to the use of e-cigarettes.	Suhling et al. [55]
Behavioral Intention	**BI1**	I am aware that e-cigarettes also have a bad effect on our health.	Alexander et al. [56]
	**BI2**	I believe that switching to e-cigarettes is a wise decision.	Cheney et al. [57]
	**BI3**	I think e-cigarette is the best alternative to traditional cigarettes.	Vandrevala et al. [58]

### 4.2. Participants selection

Data were collected from Filipino e-cigarette users to explore the behavioral drivers of e-cigarette use. The study focuses on understanding how social influence, perceived health impacts, and price sensitivity shape behavioral intentions to use e-cigarettes among Filipinos. In a country where the prevalence of e-cigarette use is rising, particularly among youth and young adults, understanding these drivers is critical for developing targeted public health interventions and regulatory strategies. Participants were identified as current e-cigarette users to ensure the relevance of their responses to the study objectives. Social influence, including peer interactions and societal norms, plays a significant role in shaping their behavior, while perceptions of health impacts and economic factors such as affordability further influence their adoption of e-cigarettes. By examining these factors, this study aims to contribute to a more comprehensive understanding of e-cigarette use in the Philippine context, providing valuable insights for effective policymaking and public health initiatives.

### 4.3. Structural Equation Modeling (SEM)

Multivariate analysis tools, particularly Structural Equation Modeling (SEM), have been widely adopted for testing hypotheses and evaluating associations between latent variables, error correlations, and factor loadings in various research studies [59–61]. More importantly, it allows for the examination of complex causal linkages among variables [62–64]. This approach combines component analysis and multiple regression analysis to explore the structural relationships between measured variables and latent constructs [65,66]. SEM has found increasing utility in scientific research, including the analysis of consumer behavior in diverse domains such as the clothing industry [67], online delivery systems [68], e-learning platforms [69] and more importantly for this study, smoker behavior [70].

## 5. Results and discussion

### 5.1. Demographic profile

From Table 2, the researcher distributed the survey to 310 Filipino participants divided according to gender, age, User of e-cigarettes, and level of education. Through frequency counts and percentages, the demographic profile of the respondents was determined. All of the participants have already used and experienced e-cigarettes, Gender-wise, males have a frequency of 126 or 40.6% of the participants. On the other hand, female participants reached 162 or 52.3%. Twenty-one participants (6.8%) opted to conceal their genders while only 1 participant (0.3%) identified as non-binary.

**Table 2:** Demographic profile.

Factor	Category	Frequency	Percent %
**Gender**	Male	126	40.6%
	Female	162	52.3%
	Prefer not to say	21	6.8%
	Others	1	0.3%
**Age**	18–24	235	75.8%
	25–40	63	20.3%
	41 and above	12	3.9%
**Users of** **e-cigarettes**	Yes No	310 0	100% 0
**Level of** **Education**	High school	46	14.8%
	College	192	62%
	Graduate/Working	67	21.6%
	Other	5	1.6%

The researcher divides the age into 9–24 years old, 25–40 years old, and 41 and above. In 18–24, the researchers gathered a frequency of 235 which represents 75.8% of the respondents. Next, 63 participants were classified under the 25–40-year bracket amounting to 20.3%. Lastly, 12 participants were at the age of 41 and above, amounting to 3.9% of the respondents. Lastly, they were sorted according to level of education namely: Junior/Senior High School, College, Graduate or Working, and Others. Forty-six (46) participants identified as Junior or Senior High School, representing 14.8%, 192 College students (62%), 67 Graduate or Working participants (21.6%), and finally with 5 participants affiliated outside these categories (1.6%).

### 5.2. Statistical analysis results

**Fig 2:**
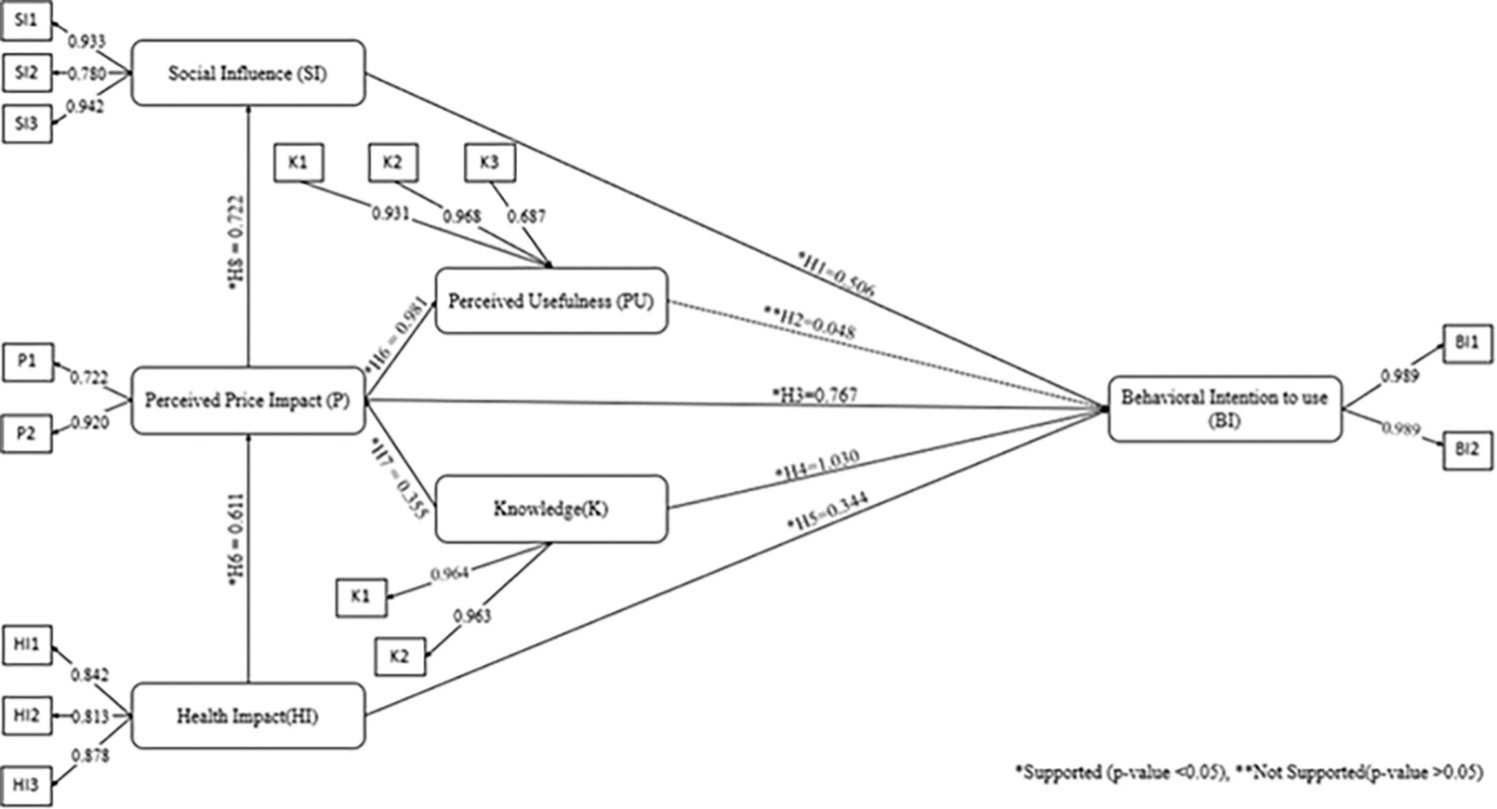
The final SEM’.

Fig 2 represents the final results of SEM and Table 3 is composed of the obtained data’s mean, median, standard deviation, and observed minimum and maximum. Mean is the sum of the values divided by the total number of values is the mean; median on the other hand is the middle number in a list of numbers in either ascending or descending order; finally, standard deviation represents how much a group of numbers diverges. The terms minimum and maximum simply describe the least and highest values in the data set, respectively.

**Table 3:** Indicators.

	Mean	Median	Observed min	Observed max	Standard deviation
**BI2**	2.994	3.000	1.000	5.000	1.849
**BI3**	2.990	2.000	1.000	5.000	1.772
**HI4**	2.145	2.000	1.000	4.000	1.122
**HI5**	2.432	3.000	1.000	4.000	1.047
**HI6**	2.287	3.000	1.000	3.000	0.879
**K2**	2.577	3.000	1.000	4.000	1.398
**K3**	2.148	2.000	1.000	4.000	1.123
**P1**	2.713	3.000	1.000	4.000	0.879
**P2**	2.571	3.000	1.000	5.000	1.290
**SI1**	2.142	3.000	1.000	3.000	0.990
**SI2**	1.287	1.000	1.000	2.000	0.452
**SI3**	2.290	2.000	1.000	4.000	1.032
**U1**	2.568	3.000	1.000	5.000	1.293
**U2**	2.426	2.000	1.000	5.000	1.290
**U3**	2.723	3.000	1.000	4.000	1.278

Social Influence has a mean value of 2.142, a median of 3.000, an observed minimum value of 1.000, an observed maximum value of 3.000, and a standard deviation value of 0.990. Perceived Usefulness has a mean value of 2.658, a median of 3.000, a minimum value of 1.000, a maximum value of 5.000, and a standard deviation value of 1.293. Perceived Price Impact have a mean value of 2.713, a median of 3.000, an observed minimum value of 1.000, an observed maximum value of 4.000, and a standard deviation value of 0.879. Knowledge has a mean value of 2.577, a median of 3.000, an observed minimum value of 1.000, an observed maximum value of 4.000, and a standard deviation value of 1.398. Health Impact has a mean value of 2.145, a median of 2.000, an observed minimum value of 1.000, an observed maximum value of 4.000, and a standard deviation value of 1.398. Behavioral Intention to use has a mean value of 2.994, a median of 3.000, an observed minimum value of 1.000, an observed maximum value of 5.000, and a standard deviation value of 1.849.

On the other hand, Table 4 shows the results for factor loadings, composite reliability, average variance extracted (AVE), and Cronbach’s alpha, which collectively provide evidence of validity and reliability for the measures used in this study. To achieve robust results, factor loadings ideally should be at least 0.7, but a minimum threshold of 0.5 is acceptable. Composite reliability should exceed 0.7, and AVE for each variable should be equal to or greater than 0.5 [71]. Items with initial loading values below 0.700 were excluded from the final analysis, as these items failed to adequately capture the variance within the variable [72]. Convergence validity was assessed by calculating the average AVE. The composite reliability values exceed the recommended threshold of 0.7, and the factor loadings surpass the minimum acceptable value of 0.5, affirming the robustness of the variables used in the study quality testing is important for assessing the reliability of data provided in the study. Cronbach’s alpha is a commonly used measure of test reliability. There are various reports of acceptable and recommendable values for alpha ranging from 0.70 to 0.95. Low alpha scores may be due to a small number of questions, low correlation between items, or heterogeneous composition. Since the result reached the acceptable value, the variables indicated are consequently well supported [73–75].

**Table 4:** Validity and reliability.

Variable	Code	Factor Loadings		Mean	StD	Composite Reliability(CR)	Average Variance Extracted	Cronbach’s Alpha
		Initial	Final					
**Behavioral Intention to use** **(BI)**	BI1	0.541	**-**	-	-	0.978	0.979	0.978
	BI2	0.981	0.989	2.994	1.849			
	BI3	0.982	0.989	2.990	1.772			
**Health Impact** **(HI)**	HI1	0.412	-	-	-	0.882	0.714	0.810
	HI2	0.522	-	-	-			
	HI3	0.587	-	-	-			
	HI4	0.788	0.842	2.145	1.122			
	HI5	0.741	0.813	2.432	1.047			
	HI6	0.821	0.878	2.287	0.879			
**Knowledge** **(K)**	K1	0.497	-	-	-	0.963	0.928	0.922
	K2	0.952	0.964	2.577	1.398			
	K3	0.957	0.963	2.148	1.123			
**Perceived Price Impact** **(P)**	P1	0.700	0.722	2.713	0.879	0.810	0.683	0.663
	P2	0.911	0.920	2.571	1.290			
	P3	0.564	-	-	-			
**Social Influence** **(SI)**	SI1	0.933	0.933	2.142	0.990	0.918	0.789	0.875
	SI2	0.779	0.780	1.287	0.452			
	SI3	0.942	0.942	2.290	1.032			
**Perceived Usefulness** **(PU)**	U1	0.931	0.931	2.568	1.293	0.902	0.759	0.839
	U2	0.967	0.968	2.426	1.290			
	U3	0.687	0.687	2.723	1.278			

Table 5 shows the Heterotrait–Monotrait Ratio of Correlations (HTMT), an average Heterotrait–Heteromethod correlation relative to average Monotrait-Heteromethod correlations. It is possible to see Heterotrait–Hetero method correlations, which are correlations of indicators across constructs measuring different phenomena, as well as Monotrait-Heteromethod correlations, which are correlations of indicators measuring the same construct, by using a correlation matrix.

**Table 5:** Heterotrait monotrait ratio.

	SI1	SI2	SI3	U1	U2	U3	P1	P2	P3	K1	K2	K3	HI1	HI2	HI3	HI4	HI5	HI6	BI1	BI2	BI3
**SI1**	1																				
**SI2**	0.56	1																			
**SI3**	0.738	0.776	1																		
**U1**	0.904	0.649	0.604	1																	
**U2**	0.798	0.437	0.445	0.806	1																
**U3**	0.411	0.676	0.741	0.356	0.541	1															
**P1**	0.396	-0.197	-0.052	0.415	0.531	0.022	1														
**P2**	0.904	0.649	0.711	0.867	0.942	0.679	0.415	1													
**P3**	-0.329	-0.195	-0.429	-0.259	-0.087	-0.284	-0.577	-0.259	1												
**K1**	0.638	0.644	0.584	0.624	0.45	0.421	0.445	0.624	-0.739	1											
**K2**	-0.613	0.003	-0.116	-0.578	-0.898	-0.271	-0.687	-0.73	0.002	-0.185	1										
**K3**	-0.647	-0.131	-0.226	-0.769	-0.754	-0.197	-0.804	-0.668	0.353	-0.362	0.773	1									
**HI1**	0.899	0.21	0.488	0.765	0.868	0.32	0.63	0.852	-0.265	0.443	-0.863	-0.766	1								
**HI2**	-0.19	-0.093	-0.043	-0.389	-0.545	-0.302	-0.152	-0.389	-0.363	0.323	0.56	0.589	-0.321	1							
**HI3**	-0.541	-0.118	-0.186	-0.562	-0.206	0.389	0.028	-0.228	-0.21	0.004	0.172	0.328	-0.419	0.163	1						
**HI4**	-0.86	-0.366	-0.55	-0.87	-0.857	-0.441	-0.709	-0.87	0.456	-0.556	0.773	0.924	-0.899	0.461	0.328	1					
**HI5**	-0.505	-0.275	-0.149	-0.379	-0.654	-0.228	-0.222	-0.596	-0.135	-0.419	0.593	0.133	-0.55	-0.079	0.058	0.297	1				
**HI6**	-0.37	-0.195	0.017	-0.433	-0.722	-0.312	-0.68	-0.594	0.22	-0.55	0.707	0.518	-0.51	0.152	-0.253	0.518	0.726	1			
**BI1**	-0.605	-0.227	-0.469	-0.375	-0.783	-0.651	-0.353	-0.768	0.101	-0.309	0.76	0.352	-0.753	0.238	-0.135	0.576	0.704	0.577	1		
**BI2**	0.972	0.43	0.58	0.903	0.876	0.325	0.5	0.903	-0.22	0.542	-0.764	-0.74	0.954	-0.31	-0.557	-0.892	-0.571	-0.478	-0.643	1	
**BI3**	0.825	0.213	0.28	0.871	0.84	0.09	0.645	0.763	-0.128	0.378	-0.829	-0.885	0.893	-0.474	-0.604	-0.885	-0.443	-0.518	-0.457	0.922	1

In this case, a more liberal criterion is 0.9; if HTMT is less than 0.90, the researchers establish this current validity. In the literature, the conservative criterion is 0.85. If HTMT is less than 0.85, discriminant validity is established. The square root of the Extracted Average Variance (AVE) is always greater than the correlation between the constructs, implying that the largest value is always in the diagonal thereby establishing discriminant validity. For the factors Health Impact, Knowledge, Social Influence, Usefulness, and Price, behavioral intention is higher than the other constructs. Through the Fornell-Locker criterion and a new method adopted from [76], discriminant validity was tested and arrived with a cross-loading of 92.20%.

To demonstrate the validity of the proposed model, a model fit study was done. Table 6 demonstrates that all parameter estimates surpassed the minimal threshold value, suggesting that the suggested model is satisfactory. The SEM fit indices were derived using goodness of fit measurements such as the NFI, and SRMRSmartPLSV.4 was used to generate these indices. Gefen [77] define 0.8 as a critical NFI value. This study yielded an acceptable NFI value of 0.810. Furthermore, according to [71,78], SRMR must be less than or equal to 0.08 (≤ 0.08) [78]. This study’s SRMR is 0.057, indicating that a lower value yielded a more favorable result. As a result, the result showed that the data was well suited to the final SEM framework.

**Table 6:** Model of fit.

Goodness of Fit	Estimates	Threshold	Reference
SRMR	0.057	<0.080	Hu L.T. & Bentler P.M. [78]
Chi-square (Adjusted)	3.124	<5.000	Hu L.T. & Bentler P.M. [78]
Normative Index (NFI)	0.810	>0.800	Baumgartner H. & Homburg C. [79] Gefen et al. [77]

Table 7 summarizes the quantified relationship of each construct with each other and behavioral intention. The results from the model reveal values in Path Coefficients and Outer Loadings. Path coefficients greater than or equal to 0.80 are indicative of convergent validity [80]. Convergent validity was established for the relationships between the five factors. Furthermore, Outer Loadings represent the estimated relationships in reflective measurement models, quantifying each item’s contribution to the respective construct. Items with outer loadings less than 0.50 are typically removed from the measurement model as they contribute relatively less to the underlying factors [81]. Hypothesis is only supported when a factor’s P value is less than 0.05 [71]. Circling back to Table 4, the researchers confirmed the satisfactory reliability and validity of the study.

**Table 7:** Structural estimates (hypothesis testing).

Hypothesis	Relationship	Original sample (O)	Sample mean (M)	Standard deviation (STDEV)	T statistics (O/STDEV)	P values	Decision
H_1_	Social Influence → Behavioral Intention to use	0.506	0.506	0.020	25.840	0.000	Supported
H_2_	Perceived Usefulness → Behavioral Intention to Use	0.048	0.050	0.032	1.487	0.137	Not Supported
H_3_	Perceived Price Impact → Behavioral Intention	0.767	0.769	0.023	33.097	0.000	Supported
H_4_	Knowledge → Behavioral Intention to Use	1.030	1.029	0.021	48.593	0.000	Supported
H_5_	Health Impact → Behavioral Intention to use	0.344	0.344	0.005	67.752	0.000	Supported
H_6_	Health Impact → Perceived Price Impact	0.611	-0.614	0.031	19.547	0.000	Supported
H_7_	Knowledge → Perceived Price Impact	0.355	0.352	0.034	10.531	0.000	Supported
H_8_	Perceived Price Impact → Social Influence	0.722	0.724	0.016	45.326	0.000	Supported
H_9_	Perceived Price Impact → Usefulness	0.981	0.931	0.005	198.987	0.000	Supported

The results reveal shown in Table 7 that Knowledge (H_4_) has the strongest impact on behavioral intention to use e-cigarettes (β = 1.030, t = 48.593, p < 0.001). This finding underscores the critical role of awareness and understanding of e-cigarette-related information in shaping users’ decisions to adopt them. Followed by perceived price impact (H_3_) significantly influence on behavioral intention (β = 0.767, t = 33.097, p < 0.001), highlighting the importance of affordability and cost considerations in users’ decision-making processes. Social influence (H_1_) is another significant factor, with a positive relationship to behavioral intention (β = 0.506, t = 25.840, p < 0.001), showing that societal norms and peer behaviors strongly shape individuals’ adoption of e-cigarettes. Additionally, Health impact (H_5_) significantly influences behavioral intention (β = 0.344, t = 67.752, p < 0.001), suggesting that users’ perceptions of health risks or benefits play an important role in their decisions. Beyond direct relationships with the behavioral intention to use e-cigarettes as an alternative to traditional cigarettes, other constructs exhibit significant interactions. Perceived price impact strongly affects perceived usefulness (H_9_: β = 0.981, t = 198.987, p < 0.001) and social influence (H_8_: β = 0.722, t = 45.326, p < 0.001), emphasizing its role in shaping perceptions and social dynamics. Health impact significantly influences perceived price impact (H_6_: β = 0.611, t = 19.547, p < 0.001), reflecting how perceived health risks may alter users’ views on affordability or value. Similarly, knowledge impacts perceived price impact (H_7_: β = 0.355, t = 10.531, p < 0.001), indicating that awareness of e-cigarette risks or benefits affects how users perceive their cost-effectiveness.

Conversely, perceived usefulness (H_2_) does not show a significant relationship with behavioral intention (β = 0.048, t = 1.487, p = 0.137). This suggests that functional benefits, such as convenience or harm reduction, are not primary drivers of e-cigarette adoption. These findings provide a hierarchy of behavioral drivers, offering valuable insights into which factors most significantly influence e-cigarette use and guiding effective public health interventions and regulatory strategies.

### 5.3 Theoretical implications

The findings of this study provide critical theoretical insights into the behavioral drivers of e-cigarette use in the Filipino context. The significant influence of social influence on behavioral intention validates social cognitive theory, emphasizing how societal norms and peer behaviors shape e-cigarette adoption. Knowledge, as the most impactful factor, reinforces the theory of planned behavior, highlighting that informed awareness about risks is essential in shaping attitudes and intentions. Perceived price impact further aligns with consumer behavior theories, demonstrating that affordability and value strongly drive e-cigarette adoption, particularly in price-sensitive populations like the Philippines. The significant role of perceived health impact supports the health belief model, confirming that perceptions of risk influence behavioral intentions and evaluations of price and value. Conversely, the non-significant relationship between perceived usefulness and behavioral intention challenges assumptions that functional benefits, such as harm reduction, are primary motivators.

### 5.4 Practical and managerial implications

The strong influence of social influence on behavioral intention highlights the critical role of societal norms and peer dynamics in shaping e-cigarette adoption. Public health campaigns should leverage this insight by countering the normalization of vaping, particularly among young adults, through targeted messaging that emphasizes its social and health risks. Similarly, the substantial impact of knowledge on behavioral intention underscores the necessity of educational interventions that enhance awareness of the harmful effects of e-cigarettes, addressing misconceptions and promoting informed decision-making. Furthermore, the strong interconnections between perceived health impact, price, and knowledge suggest that public health strategies must adopt a holistic approach, integrating these factors to address the multifaceted drivers of e-cigarette use [82]. These results underscore the need for coordinated, data-driven policies that align social, economic, and health strategies. By addressing the behavioral drivers identified in this study, policymakers and public health stakeholders can design comprehensive interventions to mitigate the rising prevalence of e-cigarette use and protect public health in the Philippines.

### 5.5 Limitations and future directions

This study did not perform separate analyses for Generation Z and Millennials due to limitations in data availability, which prevented a robust cohort-specific comparison. As a result, the findings represent the Filipino population as a whole and may not fully capture generational differences in behavioral drivers. Future research should address this limitation by collecting larger and more targeted datasets to enable generational comparisons, such as through Multigroup Analysis (MGA) [83]. Such analyses could provide deeper insights into how factors like social influence, price sensitivity, health perceptions, and other behavioral drivers vary between cohorts, offering a more nuanced understanding of e-cigarette use among Generation Z and Millennials. Furthermore, considering the rapidly evolving landscape of e-cigarette use, the researchers recommend the exploration of advanced analytical techniques, including machine learning algorithms and artificial neural networks, in future research endeavors. These innovative approaches offer the potential to unearth deeper insights into the intricate factors influencing e-cigarette adoption among various age groups. Future research can explore extensive datasets and wider populations to uncover concealed patterns and develop predictive models of shifting trends in e-cigarette usage.

## 6. Conclusion

The growing prevalence of e-cigarette use has raised significant public health concerns, particularly in countries like the Philippines, where affordability, social norms, and limited awareness contribute to its widespread adoption. This study aimed to explore the behavioral drivers influencing e-cigarette use, focusing on social influence, knowledge, perceived price impact, perceived health impact, and perceived usefulness. By employing Structural Equation Modeling (SEM), the study sought to provide a data-driven understanding of these factors and their interrelationships. A total of 310 valid responses from Filipino e-cigarette users were analyzed, ensuring data reliability, validity, and model fit through rigorous statistical testing. The modified final model revealed that 8 out of 9 hypotheses were supported, providing robust insights into the behavioral intentions behind e-cigarette use in the Filipino context. The results showed that social influence significantly predicts behavioral intention, highlighting the critical role of societal norms, peer behaviors, and collective perceptions in shaping e-cigarette adoption. This finding underscores the importance of addressing social dynamics in public health campaigns aimed at reducing vaping prevalence and Knowledge emerged as the strongest predictor, emphasizing the critical role of awareness about the risks and impacts of e-cigarettes. This aligns with the study’s objective to explore the influence of awareness on behavior, reinforcing the need for educational campaigns to correct misconceptions and encourage informed decision-making. Perceived price impact also significantly influences behavioral intention, reflecting the importance of affordability and pricing strategies in determining e-cigarette use. These findings highlight the urgency of implementing stricter pricing regulations, such as minimum pricing policies and tax increases, to curb accessibility, particularly among economically vulnerable groups. Additionally, perceived health impact was found to significantly affect both behavioral intention and perceived price impact, demonstrating the interconnectedness of cognitive and economic factors in shaping user behavior. Conversely, perceived usefulness did not show a significant relationship with behavioral intention, challenging assumptions about the importance of functional benefits, such as harm reduction or convenience, in driving e-cigarette adoption.
